# Zero-field edge plasmons in a magnetic topological insulator

**DOI:** 10.1038/s41467-017-01984-5

**Published:** 2017-11-28

**Authors:** Alice C. Mahoney, James I. Colless, Lucas Peeters, Sebastian J. Pauka, Eli J. Fox, Xufeng Kou, Lei Pan, Kang L. Wang, David Goldhaber-Gordon, David J. Reilly

**Affiliations:** 1 ARC Centre of Excellence for Engineered Quantum Systems, School of Physics, The University of Sydney, Sydney, NSW 2006 Australia; 2 Department of Physics, Stanford University, Stanford, CA 94305 USA; 3 Stanford Institute for Materials and Energy Sciences, SLAC National Accelerator Laboratory, 2575 Sand Hill Road, Menlo Park, CA 94025 USA; 4 Department of Electrical Engineering, University of California, Los Angeles, CA 90095 USA; 5 Microsoft Station Q Sydney, Sydney, NSW 2006 Australia; 6 Present Address: Department of Physics, University of California, Berkeley, CA 94720 USA; 7 Present Address: School of Information Science and Technology, ShanghaiTech University, Shanghai, 201210 China

## Abstract

Incorporating ferromagnetic dopants into three-dimensional topological insulator thin films has recently led to the realisation of the quantum anomalous Hall effect. These materials are of great interest since they may support electrical currents that flow without resistance, even at zero magnetic field. To date, the quantum anomalous Hall effect has been investigated using low-frequency transport measurements. However, transport results can be difficult to interpret due to the presence of parallel conductive paths, or because additional non-chiral edge channels may exist. Here we move beyond transport measurements by probing the microwave response of a magnetised disk of Cr-(Bi,Sb)_2_Te_3_. We identify features associated with chiral edge plasmons, a signature that robust edge channels are intrinsic to this material system. Our results provide a measure of the velocity of edge excitations without contacting the sample, and pave the way for an on-chip circuit element of practical importance: the zero-field microwave circulator.

## Abstract (web summary)

Direct measurement of edge transport in the quantum anomalous Hall effect can be made difficult due to the presence of parallel conductive paths. Here, Mahoney et al. report features associated with chiral edge plasmons, a signature of robust edge states, by probing the zero-field microwave response of a magnetised disk of Cr-(Bi,Sb)_2_Te_3_.

## Introduction

It is now understood that ferromagnetism, by lifting spin degeneracy and breaking time reversal symmetry at zero magnetic field, can transform a topological insulator (TI) into a new phase of matter that hosts chiral edge states^1–5^. The signature of this phase is the quantum anomalous Hall effect (QAHE), in which the transverse conductance of a magnetised Hall bar remains quantised in units of the conductance quantum, even in the absence of an external magnetic field^6, 7^. Experimentally, this has been demonstrated in (Bi,Sb)_2_Te_3_ using chromium^8–10^ and vanadium^11^ dopants. Given that bulk insulators and ferromagnets are commonplace at room temperature, there is optimism that the QAHE may not be limited to the cryogenic regimes of today’s experiments. A room-temperature QAHE, in which edge states propagate without dissipation, could impact some of the challenges facing current-generation high speed integrated circuits.

The presence of robust edge states in these material systems opens up the prospect that they support plasmonic edge excitations, resonant drum-modes of the electron gas that are well-known in the context of the quantum Hall effect^12–14^. Beyond their fundamental interest, the velocity of edge plasmons excitations is typically reduced compared to the speed of light, making them ideal platforms for constructing on-chip delay networks, high-impedance transmission lines, and non-reciprocal devices such as gyrators and circulators needed for quantum information processing in present low temperature setups^15–17^. Recent theoretical work has also highlighted the potential of gapped Dirac materials to host chiral plasmons at optical frequencies, arising from the non-zero Bloch band Berry curvature^18, 19^.

Here we investigate the zero-field plasmonic response of a magnetised, contactless disk of the ferromagnetic TI Cr-(Bi,Sb)_2_Te_3_. The fabrication of both Hall bars and resonant disk structures enables us to make a one-to-one comparison between the transport data and the microwave excitation spectrum of the material. By implementing a three-port circulator configuration, we show that the low-frequency plasmon response exhibits non-reciprocal behaviour, which is consistent with chiral edge plasmons. The existence of such plasmonic modes in the disk and their correspondence with a minimum in the longitudinal resistance of the Hall bar provide further convincing evidence that this system supports a robust edge state. Finally, we examine the dependence of circulation on excitation power and temperature, suggesting that microwave measurements can serve as a sensitive probe of the conditions at the edge.

## Results

### Device details

**Fig. 1:**
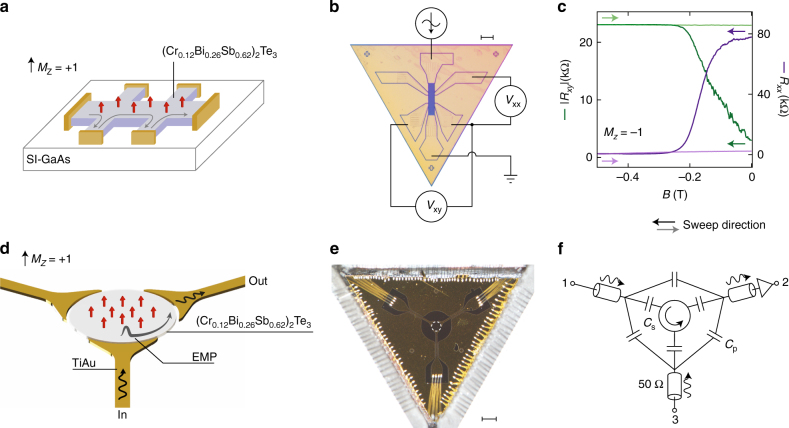
Experimental setup. **a** Illustration of the quantum anomalous Hall effect in a three-dimensional topological insulator thin film with ferromagnetic dopants. **b** Photograph of a Hall bar fabricated on a device with seven quintuple layers of (Cr_0.12_Bi_0.26_Sb_0.62_)_2_Te_3_ grown epitaxially on a GaAs substrate. Scale bar is 100 μm. Standard lock-in techniques are used to measure the transport properties of the material. **c** Transverse and longitudinal resistance (*R*_*xy*_, green, and *R*_*xx*_, purple) as the perpendicular magnetic field is swept out to −0.5 T (dark coloured lines), and then back to zero field (light shaded lines). **d** Cartoon of a three-port circulator device with a magnetic topological insulator. **e** Photograph of the circulator device, scale bar is 500 μm. **f** A circuit schematic for the experimental setup. The parasitic capacitances between port electrodes (*C*_p_) and from the ports to the plasmonic modes (*C*_s_) are indicated. Port 2 is connected to a low-noise cryo-amp operating at 4 K, allowing measurement of *S*_21_ and *S*_23_ through a common output line

Turning to the experimental setup shown in Fig. 1, the magnetic three-dimensional (3D) TI used to make the circulator and corresponding Hall bar is seven quintuple layers of (Cr_0.12_Bi_0.26_Sb_0.62_)_2_Te_3_. The film is grown on a semi-insulating (111)B GaAs substrate by molecular beam epitaxy, then capped with alumina to protect the surface. To define the microwave circulator, we use photolithography to pattern a circular, 330 μm diameter mesa and etch away the remaining film via Ar ion milling. We next pattern capacitive contacts and a ground plane, depositing 120 nm Au by e-beam evaporation. The contacts are designed to be 20 μm away from the mesa edge.

### Initial sample magnetisation

Starting at zero field, the transport data in Fig. 1c show the longitudinal and transverse resistances of the Hall bar, *R*_*xx*_ and *R*_*xy*_, during the initial magnetisation sequence, sweeping the field from zero to *B* = −0.5 T at the cryostat base temperature of *T* = 20 mK (dark purple and green lines). As the field is applied for the first time we observe *R*_*xx*_ drop from ~80 kΩ to ~500 Ω as *R*_*xy*_ increases towards *h*/*e* ^2^ (*h* is Planck’s constant and *e* the electron charge). These resistance values are found to persist after waiting several hours at −0.5 T. The field is then swept back to zero, with transport data in this direction shown as lightly-shaded lines in Fig. 1c. Over this range, we observe that *R*_*xy*_ does not vary by more than 1%, while *R*_*xx*_ increases slowly to 2.3 kΩ at *B* = 0 T. An accurate and precise measurement of *R*_*xy*_ requires accounting for possible geometric effects of the contacts and calibration using a known resistance standard, as was done in ref. ^20^. In the absence of these corrections, the measured resistance plateau value of 25,750 Ω for this device is within the uncertainty expected for the quantum of resistance, 25,813 Ω^8–10^.

We compare these transport data with the microwave response of an etched TI disk on the same material, configured as a circulator as shown in Fig. 1d–f. Similar designs comprising an rf excitation and detection port have been used to probe edge magnetoplasmons (EMPs) in the quantum Hall effect regime in GaAs semiconductors^16, 21, 22^ and in graphene^23–25^. These EMP modes are charge density waves supported by edge channels at the boundary of the material. For traditional semiconductor samples, such as GaAs, the propagation velocity and therefore microwave frequency response of the EMP is set by the ratio of the electric and magnetic fields at the edge. In the case of ferromagnetic topological insulators, the existence of a robust edge is related to the electronic band structure, and can persist in the absence of an external magnetic field.

**Fig. 2:**
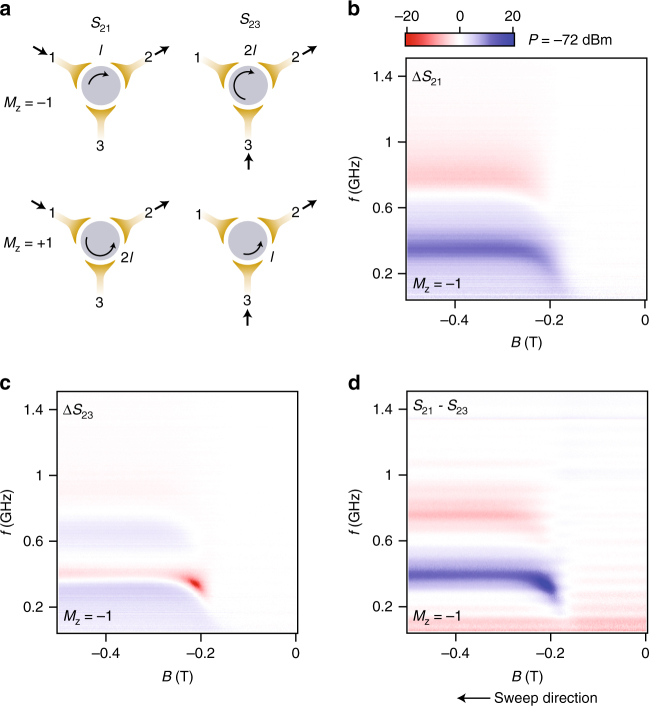
Microwave transmission measurement. **a** Illustration of chiral edge transport in a circulator setup for different magnetisation and port configurations. Arc path lengths are denoted as *l* and 2*l*. **b**, **c** show the microwave response of signals excited from ports 1 and 3, and amplified out of port 2, as the magnetic field is varied. These traces have been normalised to the reciprocal background prior to sample magnetisation, such that the colour bar represents Δ*S* in dB. In **d**, the colour bar shows *S*_21_ − *S*_23_ in dB, where the difference between the bare S-parameter traces is plotted without background normalisation. Past the coercive field of −0.16 T, strong non-reciprocity is observed at two distinct frequency bands, with opposite amplitudes

In our setup, a 3-port design further allows the non-reciprocal character of the device to be probed by determining whether the signal traverses the TI disk via a left-handed or right-handed path^15, 16^. The experiment comprises *S*-parameter measurements in which the amount of microwave power transmitted from port 1 to port 2 (*S*_21_), or port 3 to port 2 (*S*_23_) is detected as a function of external magnetic field and magnetisation state $\left( {M_z = M{\mathrm{/}}\left| M \right| = \pm 1} \right)$ of the TI disk (see Fig. 2a). The two symmetrical paths, port 1 to 2 and port 3 to 2, are designed to be equivalent in the absence of chiral transport.

We note that for any measurement, microwave power can be coupled directly and reciprocally between the ports via the geometric (parasitic) capacitance that shunts the disk (*C*_p_ in Fig. 1f). In our sample geometry *C*_p_ is estimated to be in the vicinity of a few hundred femtofarads. The combination of a direct capacitive path in parallel with the conductive edge-channels in the disk creates an interferometer in which signals travel at the speed of light in the capacitive arm, and at a velocity in the other arm that is determined by the plasmonic response^16^. It is primarily the difference in velocities (and, to a lesser extent, path lengths) between the two paths of the interferometer that yields a phase offset between the two signals when they recombine at the receiving port. Further, the amplitude of the signals is set by the impedances of the two paths; if the edge plasmon resonator has a moderate Q-factor, these amplitudes can easily be made comparable by driving the circuit at a frequency slightly detuned from the resonant mode of the disk. Within this interference picture, the response of the circuit can be interpreted as a Fano resonance that depends on the length of path travelled by the edge plasmon, *l* or 2*l* depending on the excitation port and magnetisation direction (Fig. 2a). With port 2 always set to be the receiving port, transmitting power from port 1 or port 3 thus configures the edge length to be *l* or 2*l*. The difference between the two paths’ transmission, measured as *S*_21_ − *S*_23_, determines the isolation or non-reciprocity of the circuit.

### Microwave circulator response

We begin the presentation of the circulator data in a similar manner to the transport measurements, starting at zero magnetic field and recording *S*_21_ and *S*_23_ as the external field is stepped out to −0.5 T. To enable a direct comparison between transport and the microwave response of the disk during the one-off magnetisation sequence, we acquire transport data as well as *S*_21_ and *S*_23_ at a fixed magnetic field before stepping the field (i.e., all data in Figs. 1c and 2 were obtained concurrently). All individual *S*_21_ and *S*_23_ data throughout the paper are normalised (denoted by Δ) by subtracting the initial frequency dependence of the signal in logarithmic units (dB) in the unmagnetised state, *B* = 0 and *M*_*z*_ = 0, where the response is reciprocal. This calibration trace is taken at cryostat base temperature (*T* ~ 20 mK) and a port excitation power of −72 dBm. This normalisation alleviates frequency-dependent artefacts, for instance transmission oscillations due to line impedance mismatch that do not evolve with magnetic field (Supplementary Fig. 1).

Forward transmission Δ*S* is shown in Fig. 2b, c as a function of frequency and magnetic field for the two paths *S*_21_ and *S*_23_. As the sample begins to magnetise at the coercive field (~−0.16 T), we observe resonance-like dips and peaks in the frequency spectrum of the disk, evident as red and blue coloured horizontal bands appearing at the field strength where *R*_*xy*_ (Fig. 1c) approaches the resistance plateau *h*/*e* ^2^. This is the microwave signature of the QAHE. Compared with EMPs in 2-dimensional electron systems^16^ where the frequency *ω*_EMP_ is proportional to 1/*B*, in the TI we observe a flat frequency band as a function of magnetic field, centred at the fundamental mode of the edge plasmon, near 400 MHz. This is consistent with dc transport measurements of the Hall resistance, which takes on a constant, quantised value after the sample is magnetised. Measuring the frequency at which these resonances occur in combination with the circumference of the TI disk gives a velocity at the fundamental mode of ~4 × 10^5^ m s^−1^, similar to what is found in other structures comprising stacks of semiconductors^16, 21, 22^.

The microwave response shows that the parameters *S*_21_ and *S*_23_ deviate from each other as the disk becomes magnetised. This is a result of the non-reciprocity of the system, evident in Fig. 2d, where we have subtracted the bare *S*-parameters (*S*_21_ − *S*_23_) from each other to show the difference between the two configurations of the circulator. Again, we interpret these measurements of *S*_21_ and *S*_23_ as characterising paths around the edge of the disk in the same (chiral) direction with arc-length *l* and 2*l* (Fig. 2a). Considering the measurement in Fig. 2d, it is apparent that microwave power can both circulate near the fundamental edge plasmon frequency (blue frequency band) and counter circulate in an opposite direction near the first harmonic (red frequency band). This behaviour is also observed for GaAs devices in the quantum Hall regime^16^ and is understood to arise from a Fano-like interference between the slow-velocity resonantly circulating edge mode and the parallel capacitive path^26^. We remark that the observation of circulation and counter-circulation is a further signature of the plasmonic response of the chiral edge state.

### Sweeping the magnetic field

**Fig. 3:**
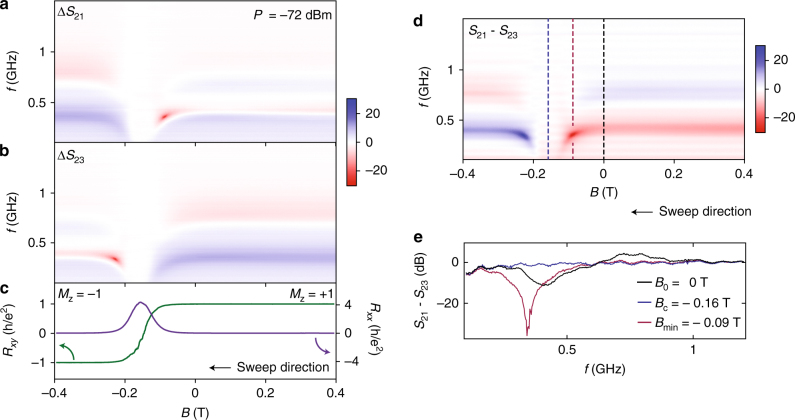
Rf and dc response with magnetic field. **a**, **b** Normalised Δ*S*_21_ and Δ*S*_23_ responses measured concurrently with the dc transport data in **c**. A pre-magnetisation frequency-dependent background has been subtracted from each of the traces in **a**, **b**. The colour scale represents Δ*S* (dB). **c** Transverse (green) and longitudinal (purple) resistances measured on a Hall bar as the magnetisation direction is swept from positive to negative. Away from the coercive field, raw *R*_*xy*_ approaches the resistance quantum while *R*_*xx*_ measures ~500 Ω. In **d** we compare the difference between the bare *S*_21_ and *S*_23_ paths (indicated by the colour scale in dB), providing a measure of isolation in the system. **e** Line cuts through **d** at zero applied magnetic field (*B*_0_, black line), the coercive field (*B*_c_, blue) and at the point where a power minimum is observed (*B*_min_, mauve)

The quantum anomolous Hall effect is unique in that it supports a chiral edge state at zero applied field^20^. To examine the zero-field response of the magnetised TI system, we continue to take transport measurements on the Hall bar concurrent with *S*-parameter data on the circulator, as the system is swept from positive to negative field through zero, as shown in Fig. 3a, b. The transport data in Fig. 3c show the familiar signature of the QAHE with *R*_*xx*_ peaking and *R*_*xy*_ switching sign at the coercive field indicated by the blue dashed line (~−0.16 T). At *B* = 0, the system remains magnetised with *R*_*xy*_ reaching a maximum value of 25.75 kΩ.

In comparison to the transport measurements, the microwave response of the TI reveals new information. The response of the disk for each of the signal configurations, characterised by Δ*S*_21_ in Fig. 3a and Δ*S*_23_ in Fig. 3b, is strongly asymmetric about the coercive field. Symmetry is restored, however, if in addition to the sign of the magnetisation, the ports are also interchanged, so that the red band in Δ*S*_21_ on the left of the coercive field mirrors the red band in Δ*S*_23_ on the right, and vice versa for blue features. This strong non-reciprocity is most evident in the differential form of the data *S*_21_ − *S*_23_, as shown in Fig. 3d. At zero field, the circulator continues to exhibit non-reciprocity ~10 dB (Fig. 3e). Intriguingly, the device is maximally non-reciprocal at a field approaching the coercive field, producing a hot-spot feature in the *S*-parameter response (mauve dashed-line Fig. 3d). As described below, we suggest these features are linked to enhanced dissipation.

### Power and temperature dependence

**Fig. 4:**
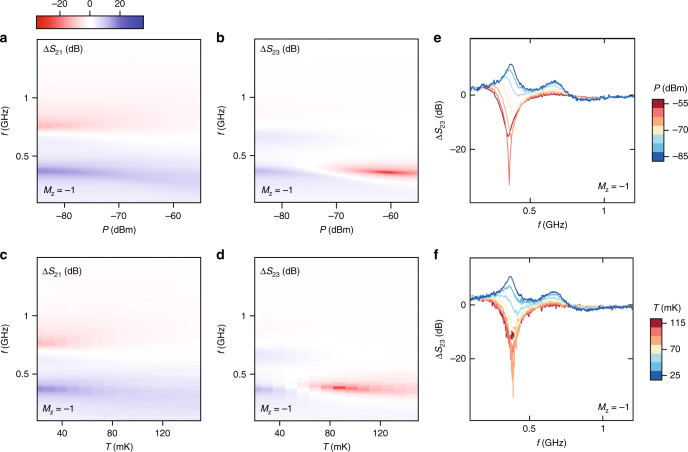
Effect of temperature and microwave power. **a**, **b** Frequency response Δ*S*_21_ and Δ*S*_23_ with increasing microwave power applied to the excitation port. For these measurements *T* ~ 20 mK. In **c**, **d** the dependence of Δ*S*_21_ and Δ*S*_23_ is measured as a function of cryostat temperature while the applied port power is kept low at −87 dBm. In **a**–**f**, all the data are taken at zero applied magnetic field after the sample has been magnetised with *M*_*z*_ = −1. Akin to Figs. 2 and 3, the same pre-magnetisation background taken at constant power and cryostat base temperature is subtracted from each of the 2D plots. The colour scale represents Δ*S* (dB). **e**, **f** 1D cuts through the colour plots of Δ*S*_23_ in **b**, **d**. The colour bar in **e** shows power (dB), while the colour bar in **f** indicates temperature (mK)

Finally, we investigate the temperature and microwave power dependence of the edge plasmon spectra in an effort to better understand the details of the zero-field edge state. At *B* = 0 and with the TI magnetised (*M*_*z*_ = −1), Δ*S*_21_ and Δ*S*_23_ are measured as a function of applied microwave power *P*, as shown in Fig. 4a, b. In addition to the usual non-reciprocity at constant power, we observe an evolution in the non-reciprocal features with increasing *P* that is dependent on the length of the edge segment. While the response of Δ*S*_21_ (characterised by arc-length *l*) begins to fade out at high powers, the amplitude of Δ*S*_23_ (2*l*) changes sign near the fundamental frequency and exhibits a pronounced minimum or hot-spot near *P* = −60 dBm. Interchanging the ports and repeating the measurement at *M*_*z*_ = +1 and *B* = 0 produces similar features in accordance with a reversal of chirality (Supplementary Fig. 2). Mirroring the dependence with power, increasing the cryostat temperature also produces a change of sign relative to the pre-magnetised state for the longer edge path (2*l*): raising *T* as in Fig. 4c, d leads to Δ*S*_21_ becoming gradually washed out, while Δ*S*_23_ produces a hot-spot around *T* = 85 mK. This effect is further illustrated in Fig. 4, where 1D cuts at constant power (e) or temperature (f) are shown for Δ*S*_23_.

## Discussion

So-called hot-spots—characterised by a strong decrease in the microwave power transmitted between ports—occur at particular magnetic fields, powers, or temperatures. Appealing to the phenomenology of the interferometer pictured in Fig. 1f, we note that the direction of power transmission between ports, either circulation or counter-circulation, is set by the relative phase and amplitude of the signals in the edge-state arm compared to the direct capacitive arm of the interferometer. In general, this picture accounts for the constructive interference of signals for the shorter edge path (*l*) and destructive interference for the longer (2*l*), when driving near resonance of the edge plasmon fundamental mode.

Extending this picture to include dissipation, we suggest that losses in either arm of the interferometer can adjust the relative amplitudes. As noted above, the relative phase of the two paths is naturally tuned by *π* upon crossing the edge plasmon resonance. At a frequency where the relative phase is *π* we might expect a sign change in the relevant *S*-parameter response. In this way, the hot-spots can be understood as regions where almost perfect cancellation between the two arms occurs. The appearance of dissipation in either path with increasing temperature, microwave power, or near the coercive field is perhaps not unexpected. Accounting for the microscopic mechanisms underlying such dissipation remains an open challenge^20, 27–32^, given that all measurements are well below both the Curie temperature of these ferromagnetic TIs (of order 10 to 20 K) and the energy scale of the exchange gap as measured by ARPES (of order 10 meV). We note that the hot-spot onset temperature of 85 mK is consistent with experimentally determined activation gaps on similar growths^20^, although we cannot rule out the possibility of parallel or counter-propagating paths.

To conclude, we have probed the plasmonic edge spectrum of a magnetic topological insulator, comparing its microwave response to the transport data. The measurement setup can be understood as an interferometer with the disk of TI in one arm of the interferometer and a parasitic capacitance in the other. Within this picture, the response of the system exhibits resonances that can be explained by accounting for the slow velocity of edge plasmons as they traverse an arc-length of the TI disk’s edge rather than the bulk. In addition to the device examined, we have studied a second circulator, fabricated from a separately-grown wafer, and found it to exhibit strikingly similar behaviour in all aspects (see Supplementary Fig. 3). We suggest that this similarity between devices is related to the robust properties of the edge state, set by the non-trivial topology of the material system rather than, for instance, the specific configuration of microscopic disorder. Taken together, our microwave measurements provide strong evidence that this material system indeed supports robust, chiral edge states at zero magnetic field, opening the prospect of compact microwave components based on magnetic topological insulators.

## Methods

### Fabrication details

The film used to make the circulator and corresponding Hall bar is seven quintuple layers of (Cr_0.12_Bi_0.26_Sb_0.62_)_2_Te_3_. We use photolithography to pattern a circular mesa with a diameter of 330 μm. We bake the Megaposit SPR 3612 photoresist at 80 °C (to avoid possible damage to the film from overheating), and develop with MF CD-30 after exposure. To define the mesa, we etch the surrounding film via Ar ion milling. After patterning the contacts and ground plane with the same photolithographic procedure, we deposit a 5 nm Ti sticking layer followed by 120 nm Au using e-beam evaporation. The capacitive contacts are designed to be 20 μm from the edge of the circular mesa. For the primary device discussed in this work, the relative misalignment of the mesa and contacts is approximately 5 μm.

### Data availability

The data that support the findings of this study are available from the corresponding authors upon reasonable request.

## Electronic supplementary material

Supplementary Information
