# Supplementary material for: Zero-field edge plasmons in a magnetic topological insulator
Source: Nat Commun. 2017 Nov 28;8:1836. doi: 10.1038/s41467-017-01984-5 (PMC5705665; doi:10.1038/s41467-017-01984-5)
Supplement: Supplementary file 1 — Supplementary Information [file 41467_2017_1984_MOESM1_ESM.pdf]

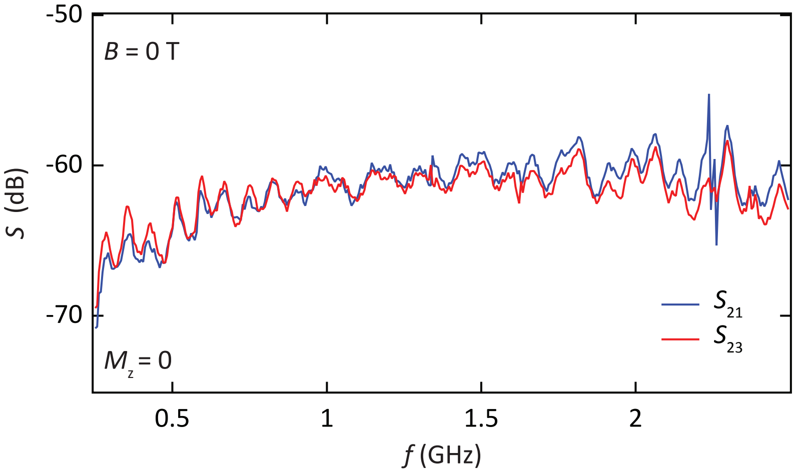

Supplementary Figure 1: Microwave transmission prior to sample magnetisation. S-parameter transmission measurements taken prior to device magnetisation at cryostat base temperature of $\sim$ 20 mK, and applied port power of -72 dBm. Traces have been corrected for amplification and attenuation added to the setup in order to provide a measure of insertion loss. Subtracting the bare S-parameter responses S_{23} from S_{21} (as shown in Fig. 2(d) of the main manuscript) yields a small residual response at $B = 0$ about 0 dB, attributed to slight differences in the line impedances of the two rf setups.

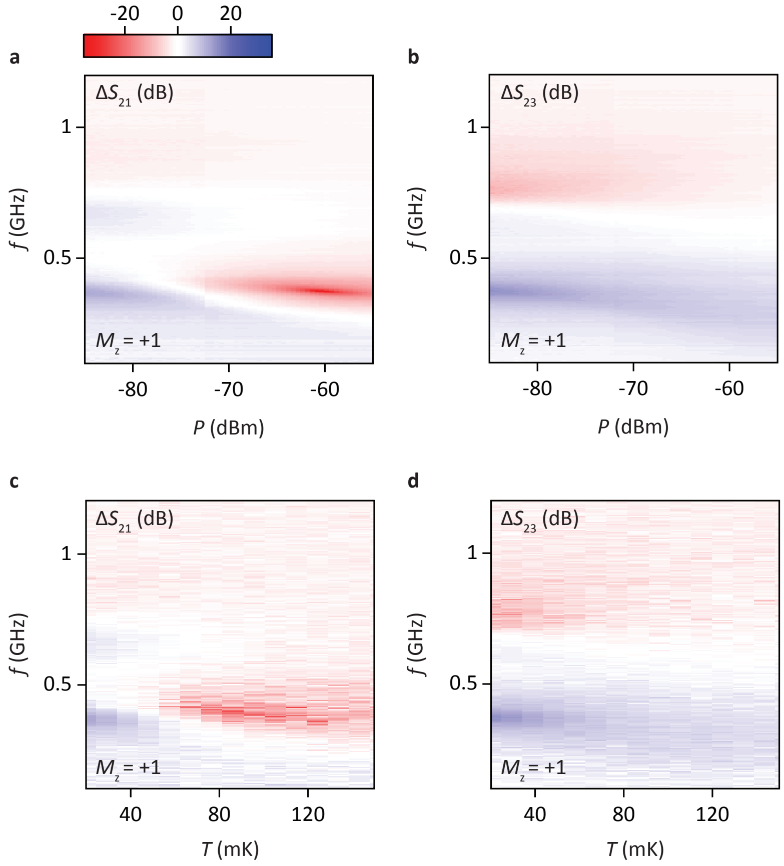

Supplementary Figure 2: Power and temperature dependence at $M_z = +1$ **:** (a) - (d) Effect of cryostat temperature and applied microwave port power on S_{21} and S_{23} at $B = 0$ once the sample has been magnetised in the positive direction, $M_z = +1$. The direction of magnetisation has been reversed with respect to the data in Fig. 4 of the main manuscript. In accordance with a reversal of chirality, hot-spots are observed in the normalised ΔS_{21} plots with both power and cryostat base temperature, corresponding to the $2l$ path in this configuration. Colour bar represents ΔS (dB).

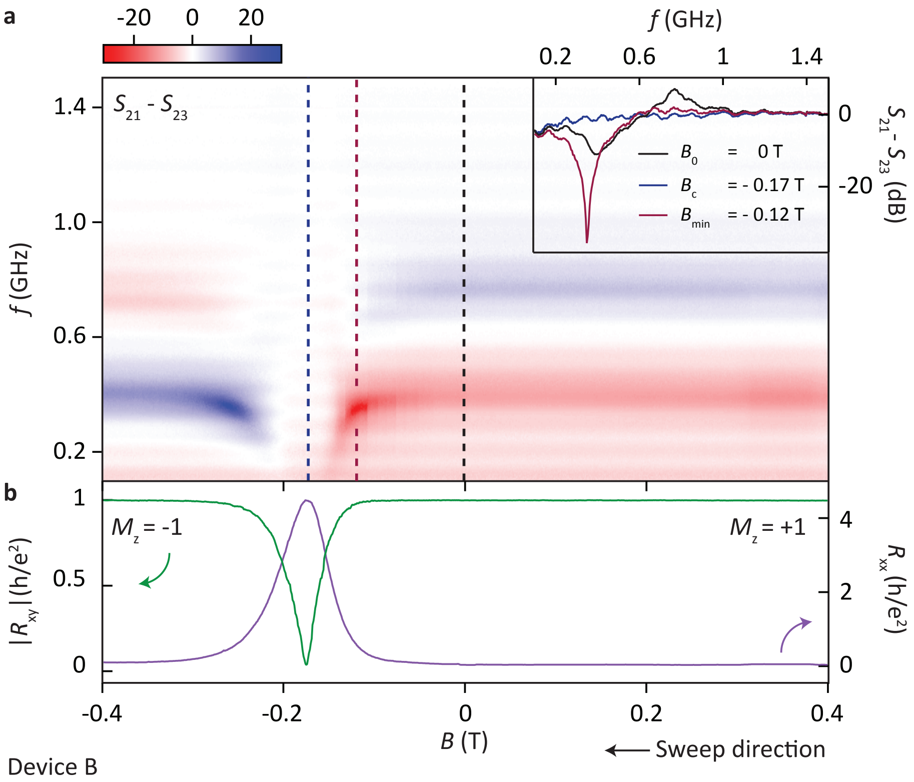

Supplementary Figure 3: Secondary device. S_{21}-S_{23} microwave spectrum is shown in (a), while Hall bar transport measurements are presented in (b) for a secondary device on a separate growth. Colour bar shows S_{21}-S_{23} in dB. The material comprises 8 quintuple layers of $(Cr_{0.12}Bi_{0.26}Sb_{0.62})_2Te_3$, with fabrication methods and circulator geometry nominally identical to the device outlined in the main manuscript. Inset shows cuts taken at constant magnetic field values corresponding to dashed lines in (a).
